# Effects of Crossbreeding on Growth Performance and Carcass Traits in Sheep Under Extensive Pasture Systems

**DOI:** 10.3390/ani16142167

**Published:** 2026-07-13

**Authors:** Bolatbek Ateikhan, Zhanat Titanov, Nadezhda Burambayeva, David Arney, Toktar Bexeitov, Alma Temirzhanova, Rustem Abeldinov

**Affiliations:** 1 Department of Zootechnology and Veterinary Medicine, Toraighyrov University, Lomov 64, 140000 Pavlodar, Kazakhstan; bolatbek_ateihanuly@mail.ru (B.A.); 07041963@mail.ru (N.B.); bexeitov.t@tou.edu.kz (T.B.); alma.temirzhanova.74@mail.ru (A.T.); abrustem@mail.ru (R.A.); 2 Institute of Veterinary Medicine and Animal Sciences, Estonian University of Life Sciences, Kreutzwaldi 1, 51014 Tartu, Estonia; david.arney@emu.ee

**Keywords:** crossbreeding, growth performance, carcass traits, meat productivity, meatiness coefficient, fat-tailed sheep, extensive pasture systems

## Abstract

Improving meat productivity of sheep under extensive pasture systems is essential for food security in arid regions. This study evaluated the effects of crossbreeding on growth performance and carcass traits of ram lambs raised exclusively on natural pastures without supplementary feeding in the Pavlodar and Abai regions of Kazakhstan. Four genotypic groups were studied: purebred Kazakh Coarse-wooled (CW × CW, *n* = 30), Meat Merino × Kazakh Coarse-wooled (MM × CW, *n* = 30), purebred Kazakh Semi-coarse-wooled (SCW × SCW, *n* = 30), and Kazakh Meat-Wool × Kazakh Semi-coarse-wooled (MW × SCW, *n* = 30). Live weights and growth performances were recorded from birth to 4–4.5 months of age. A sample slaughter of five ram lambs per group was conducted to evaluate carcass characteristics and meatiness coefficients. Data were analyzed using one-way ANOVA followed by Tukey’s HSD test. Crossbred lambs demonstrated significantly higher growth performance compared with purebred counterparts. Pre-slaughter live weight and carcass weight were highest in crossbred groups, while dressing percentage reached 54.0% (*p* < 0.001). The highest meatiness coefficient was observed in MW × SCW lambs (5.15), indicating superior carcass composition. Crossbred lambs showed a 20% increase in carcass weight and a 21–44% higher meatiness coefficient compared with purebred groups. The MW × SCW genotype exhibited the best overall performance. These findings indicate that crossbreeding is a practical genetic strategy for improving growth performance, carcass traits, and meat productivity under low-input pasture-based systems and may be applicable to sheep production in similar arid and semi-arid regions.

## Simple Summary

Sheep farming is an important sector of agriculture in Kazakhstan, but increasing meat production remains a pressing challenge. According to FAO estimates, more than 60% of the world’s sheep population is kept in extensive pasture systems where supplementary feeding is economically unfeasible. This study compared growth performance, slaughter characteristics, and carcass composition of purebred and crossbred ram lambs raised on natural pastures in the Pavlodar and Abai regions of Kazakhstan. The lambs were kept exclusively on pasture without any supplementary feeding. Four groups of lambs were evaluated: Kazakh Coarse-wooled (CW), Kazakh Semi-coarse-wooled (SCW), and their crosses with Meat Merino (MM) and Kazakh Meat-Wool (MW) breeds, respectively. A sample slaughter of five ram lambs from each group was conducted at 4–4.5 months of age to assess their meat quality characteristics. Crossbred ram lambs demonstrated significantly higher live weight, average daily gains, and meat-to-bone ratio compared to their purebred counterparts (*p* < 0.001). The highest meat yield was observed in SCW × MW crosses (83.8% of carcass weight) with a meatiness coefficient of 5.15. These results demonstrate that crossbreeding is an effective genetic strategy for improving meat production in low-input pastoral systems.

## 1. Introduction

Sheep farming is one of the most traditional and economically significant branches of animal husbandry in arid and semi-arid regions of the world. According to FAO data, the global sheep population exceeds 1.2 billion head, with more than 60% kept in extensive pasture systems prevalent in developing countries of Asia, Africa, and Latin America [1,2]. In these regions, sheep farming enables food security, rural incomes, and the sustainable use of lands unsuitable for crop production [3]. The transition to a market economy and growing urban populations create demand not only for increased production volumes but also for improved quality of meat products that meet modern standards [4]. Modern sheep farming focuses on the comprehensive improvement of breeding and production qualities through targeted selection, optimization of feeding and management, and the introduction of advanced reproductive technologies [5]. Among these approaches, crossbreeding has emerged as one of the most effective genetic strategies for improving meat productivity while preserving the adaptive capacity of indigenous breeds under extensive pasture-based production systems.

### Global Context: Sheep Farming in Arid Regions

Fat-tailed sheep breeds are an adaptive type formed as a result of long-term natural and artificial selection under continental climate conditions existent in Eurasia. Representatives of this group are distributed across a wide belt from Eastern Europe through Kazakhstan, Mongolia, and Northern China to Iran, Turkey, and the Horn of Africa [6,7]. Common features of these breeds include high adaptability to extreme temperatures, drought tolerance, and the ability to efficiently utilize sparse pasture vegetation [8].

Most local breeds in Central Asia are characterized by moderate meat productivity and pronounced fat deposition in the tail (fat tail)—a trait that reduces dressing percentage and consumer satisfaction in the modern market [9]. In conditions where the introduction of intensive feeding technologies is limited by economic and logistical factors, crossbreeding with terminal meat breeds represents one of the most accessible and effective genetic strategies [10].

Similar approaches have already been successfully applied in other regions of the world. For example, crossing Awassi with Dorper in Jordan, Merino with Dorper in South Africa, and Barbarine with exotic meat breeds in Tunisia showed improvements in dressing percentage by 3–8% and muscle tissue yields by up to 86% [11,12,13]. These studies demonstrate that crossbreeding is a practical genetic strategy for improving meat production under extensive grazing systems. However, data on the effectiveness of crossbreeding in the continental climate of the Eurasian steppe remain limited, which underscores the relevance of this study. In Kazakhstan, the predominant production system is based on extensive pasture management, where lambs are raised on natural pastures with minimal or no supplementary feeding [14]. Pasture monitoring in the study region (Pavlodar and Abai regions) revealed high floristic diversity (292 plant species from 51 families), with 39.4% of these being forage species. However, unregulated grazing has led to systematic degradation of the vegetation cover, reduced protective cover, and a shift in dominant plant species from feather-grass–fescue communities to wormwood–fescue and weed species. Under such conditions, the choice of an appropriate genetic strategy becomes particularly important for improving productivity.

Meat productivity is a key indicator of the commercial value of sheep and reflects their ability to achieve rapid growth and produce carcasses of desirable weight and quality within defined production periods [15]. This encompasses both quantitative characteristics (live weight, carcass weight, dressing percentage) and qualitative aspects of meat quality [16].

Purebred breeding ensures the stability of inherited traits, predictability of production qualities, and opportunities for pedigree selection [17]. However, crossbreeding is increasingly used to produce animals that combine the desirable traits of specialized meat sire breeds with those of highly adapted maternal lines [18]. The main advantage of crossbreeding lies in the expression of heterosis, resulting in increased viability, faster growth rates, and improved slaughter characteristics of the offspring [19]. The main components of carcass commercial value are muscle tissue, fat tissue (subcutaneous, internal, and intramuscular), and bone tissue [20]. The meatiness coefficient (muscle-to-bone ratio) serves as an objective indicator of meat productivity, reflecting the proportion of edible to inedible parts of the carcass [21].

In Kazakhstan, the Kazakh Coarse-wooled (CW) and Kazakh Semi-coarse-wooled (SCW) breeds form the basis of the national sheep population. These breeds are well adapted to local climatic conditions but can benefit from crossbreeding as an effective genetic strategy using specialized meat breeds such as Meat Merino (MM) and Kazakh Meat-Wool (MW) [22].

The Pavlodar and Abai regions are located in northeastern Kazakhstan and are characterized by a sharply continental climate with hot, dry summers and cold winters. The landscape is dominated by steppe and semi-arid pasture ecosystems that form the basis of extensive sheep production systems. The aim of this study was to evaluate and compare growth performance, slaughter characteristics, and morphological carcass composition of purebred and crossbred ram lambs raised on natural pastures without supplementary feeding in these regions and to generalize the findings in the context of global challenges in meat sheep production in arid regions.

## 2. Materials and Methods

### 2.1. Ethical Statement

All experimental procedures were conducted in accordance with the guidelines of the Ministry of Agriculture of the Republic of Kazakhstan and were approved by the Ethics Committee for the Evaluation of Scientific Research of Toraighyrov University (Pavlodar, Kazakhstan) (Approval No. 22, dated 18 June 2026). The study was conducted on commercial sheep farms under routine husbandry conditions. All animals received standard veterinary care throughout the experiment and were managed in accordance with national animal welfare regulations.

### 2.2. Experimental Design and Animals

The study was conducted on two commercial sheep farms in Kazakhstan: LLP “KH Ak Bas-Pavlodar” (Pavlodar region, 52.29° N, 77.68° E) and “KH Bepen” (Abai region, 50.61° N, 79.58° E). Animals were classified into bonitation classes according to a standard sheep breeding evaluation system based on exterior traits, live body weight, and wool productivity. The system includes elite and Class I categories, with elite representing the highest breeding level and Class I the subsequent level.

The sample size of 30 ram lambs per group was selected in accordance with common practice in animal breeding experiments and was considered sufficient to detect biologically meaningful differences in growth and carcass traits under commercial field conditions.

**Figure 1:**
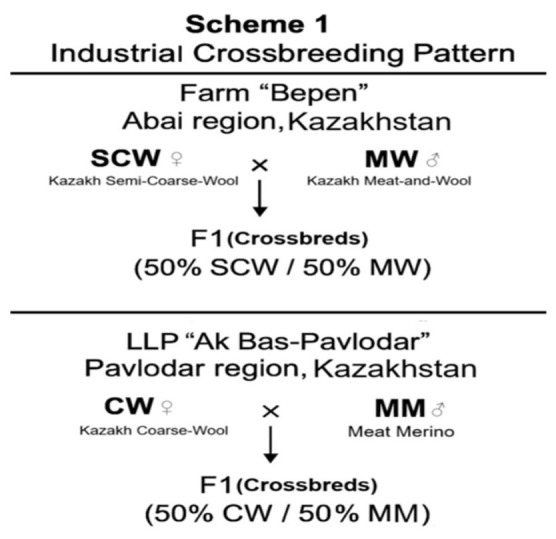
Industrial crossbreeding scheme.

**Figure 2:**
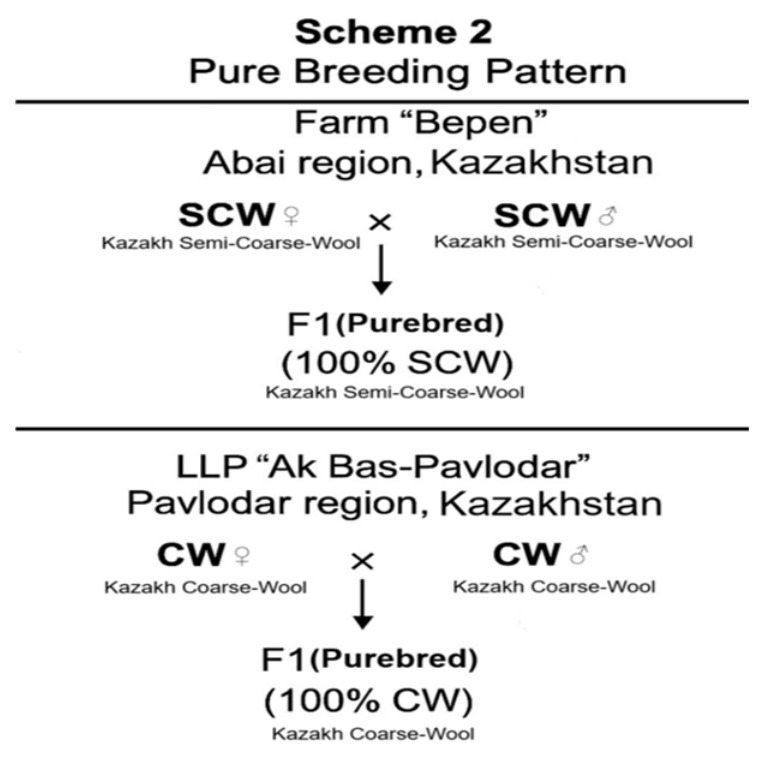
Pure breeding scheme.

A total of 120 ram lambs from four breed groups (*n* = 30 per group) were included in the study:

1. Purebred Kazakh Coarse-wooled (CW × CW): Class I ewes inseminated with Elite CW rams.
2. Kazakh Coarse-wooled × Meat Merino (MM × CW): Class I CW ewes inseminated with Elite Meat Merino rams.
3. Purebred Kazakh Semi-coarse-wooled (SCW × SCW): Class I SCW ewes inseminated with Elite SCW rams.
4. Kazakh Semi-coarse-wooled × Kazakh Meat-Wool (MW × SCW): Class I SCW ewes inseminated with Elite MW rams (Figure 1 and Figure 2).

Each crossbreeding scheme was evaluated within a single commercial farm under the same management conditions as its corresponding purebred control. Consequently, the experimental design was intended primarily to compare each crossbred group with its respective purebred control within the same farm. Comparisons between breed groups located on different farms should therefore be interpreted with caution because environmental and farm-management effects cannot be completely separated from genotype effects.

### 2.3. Pasture Conditions and Feeding

Lambs were kept exclusively on natural pastures without supplementary feeding throughout the experimental period. This approach reflects real-world conditions of sheep farming in most arid regions of the world, where the use of concentrated feeds is limited by producers’ economic capabilities [23].

Pasture vegetation was identified during this study and characterized by high floristic diversity (292 species from 51 families), with dominance of the families Asteraceae, Poaceae, Fabaceae, and Chenopodiaceae. Due to unregulated grazing, degradation of pastures was observed, manifested in reduced projective cover and a shift from feather-grass–fescue communities to less productive wormwood–fescue and weedy associations. Average pasture yield varied from 0.3 to 1.8 c/ha on heavily degraded areas to 3–5 c/ha on improved meadows.

The botanical composition and productivity of the pastures were evaluated; however, the chemical composition of the forage (crude protein, fiber fractions, and metabolizable energy) was not determined because the study was primarily designed to assess the effects of genotype under practical commercial grazing conditions.

### 2.4. Growth Performance Measurements

Live weight (LW) was determined by individual weighing using AOTE electronic platform scales (maximum capacity 350 kg, accuracy ±0.1 kg; AOTE, China) during the designated experimental periods:

- At birth;
- At 4–4.5 months of age (weaning).

The following parameters were calculated:

Absolute weight gain (ΔM): ΔM = M_2_ − M_1_

Average daily gain (ADG): ADG = (M_2_ − M_1_)/t

where M_2_ is final live weight (kg), M_1_ is initial live weight (kg), and t is the duration of the period (days).

### 2.5. Sample Slaughter and Carcass Evaluation

A sample slaughter was performed at 4–4.5 months of age using five clinically healthy ram lambs with the highest live body weight selected from each experimental group. The same selection criteria were applied across all genotype groups to evaluate the slaughter characteristics of the best-developed animals representing the productive potential of each genotype. The following parameters were recorded:

- Pre-slaughter live weight (kg);
- Hot carcass weight (kg);
- Slaughter weight (kg);
- Fat tail weight (kg) and percentage;
- Internal fat weight (kg) and percentage.

### 2.6. Carcass Morphological Composition

After slaughter, carcasses underwent anatomical dissection to determine morphological composition:

- Muscle tissue weight (kg) and percentage;
- Bone tissue weight (kg) and percentage;
- Fat tissue weight (kg) and percentage.

The meatiness coefficient (K_m_) was calculated using the formula

K_m_ = M_m_/M_k_

where M_m_ is muscle tissue (meat) weight (kg), M_k_ is bone weight (kg).

### 2.7. Statistical Analysis

Data are presented as mean ± standard deviation (SD). Statistical analysis was performed using one-way analysis of variance (ANOVA) to evaluate differences among the four genotypic groups. The primary ANOVA calculations, including sums of squares, mean squares, F-values, and significance levels, were performed in Microsoft Excel using standard statistical formulas. The results were subsequently verified using the online statistical calculator ASTATSA (https://astatsa.com, accessed on 2 July 2026), which was also used to perform Tukey’s Honestly Significant Difference (HSD) post hoc test for multiple pairwise comparisons. Statistical significance was accepted at *p* < 0.05, with additional significance levels reported as *p* < 0.01 and *p* < 0.001 where appropriate.

## 3. Results

### 3.1. Growth Performance

One-way ANOVA identified significant differences in birth weights between groups (F = 34.13, *p* < 0.001). The dynamics of live weights from birth to 4–4.5 months are presented in Table 1.

The data presented in Table 1 demonstrate a significant effect of genotype on early growth performance of lambs.

At birth, significant differences were observed among genotypes (*p* < 0.05), with the highest live weight recorded in MW × SCW lambs (5.03 ± 0.23 kg), while the lowest value was found in MM × CW lambs (4.30 ± 0.41 kg). These differences indicate a clear genetic influence on early developmental traits.

However, no statistically significant differences were detected in live weight at 4–4.5 months of age (F = 2.39; *p* = 0.072), as all genotypes were assigned the same significance group, suggesting a convergence of body weight among groups during the rearing period.

A similar pattern was observed for growth traits. The highest absolute weight gains were recorded in SCW × SCW (33.92 ± 2.25 kg) and MM × CW (33.90 ± 3.02 kg) lambs, whereas the lowest gain was observed in CW × CW animals (32.00 ± 1.86 kg). Average daily gain ranged from 267 to 283 g, with the highest values observed in SCW × SCW (283 ± 19 g) and MM × CW (282 ± 32 g) groups.

One-way ANOVA revealed a statistically significant effect of genotype on absolute weight gain (F = 2.98; *p* = 0.034) and average daily gain (F = 2.98; *p* = 0.034). Tukey’s post hoc test indicated that SCW × SCW and MM × CW groups exhibited significantly higher growth performance compared with CW × CW, while MW × SCW showed intermediate values with partial overlap.

Overall, crossbred lambs showed higher absolute and average daily weight gains than the purebred CW group, whereas no statistically significant differences were observed in final live weight at 4–4.5 months among the evaluated genotypes.

### 3.2. Slaughter Characteristics

**Figure 3:**
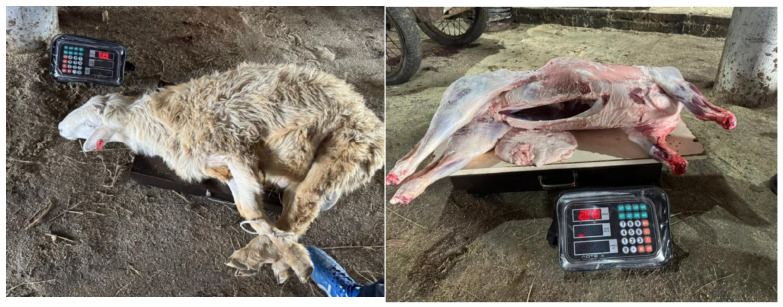
Determination of pre-slaughter weight and post-slaughter weight.

Significant differences were found for all slaughter parameters (*p* < 0.001) by breed. Slaughter data are presented in Table 2 and Figure 3.

Pre-slaughter live weight differed significantly among the genotype groups (one-way ANOVA: F (3,16) = 15.52; *p* < 0.001). The highest pre-slaughter live weight was recorded in MW × SCW lambs (46.0 ± 2.17 kg), followed by MM × CW (43.5 ± 1.61 kg), SCW (42.5 ± 1.04 kg), and CW (39.4 ± 1.10 kg). According to Tukey’s post hoc test, the MW × SCW genotype had a significantly higher pre-slaughter live weight than the CW group (*p* < 0.05), whereas MM × CW showed intermediate values and did not differ significantly from either SCW or MW × SCW.

Carcass weight was also significantly affected by genotype (one-way ANOVA: F (3,16) = 11.49; *p* < 0.001). The greatest carcass weight was observed in the MW × SCW group (24.0 ± 0.72 kg), followed by MM × CW (23.0 ± 1.22 kg), while lower values were recorded in the purebred SCW (21.0 ± 1.44 kg) and CW (20.0 ± 1.30 kg) groups. Tukey’s post hoc test demonstrated that the crossbred groups produced significantly heavier carcasses than the purebred animals (*p* < 0.05).

Slaughter weight likewise differed significantly among genotypes (one-way ANOVA: F (3,16) = 15.14; *p* < 0.001). The highest slaughter weight was obtained in MW × SCW lambs (24.8 ± 0.70 kg), followed by MM × CW (23.5 ± 1.20 kg), whereas SCW (21.2 ± 1.45 kg) and CW (20.3 ± 1.25 kg) exhibited significantly lower values. The Tukey post hoc test confirmed the significant advantage of the crossbred genotypes over the purebred groups (*p* < 0.05).

Internal fat weight was also influenced by genotype (one-way ANOVA: F (3,16) = 72.43; *p* < 0.001). The highest internal fat weight was recorded in the MW × SCW group (0.80 ± 0.07 kg), followed by MM × CW (0.50 ± 0.07 kg), whereas considerably lower values were observed in CW (0.30 ± 0.07 kg) and SCW (0.25 ± 0.05 kg).

**Figure 4:**
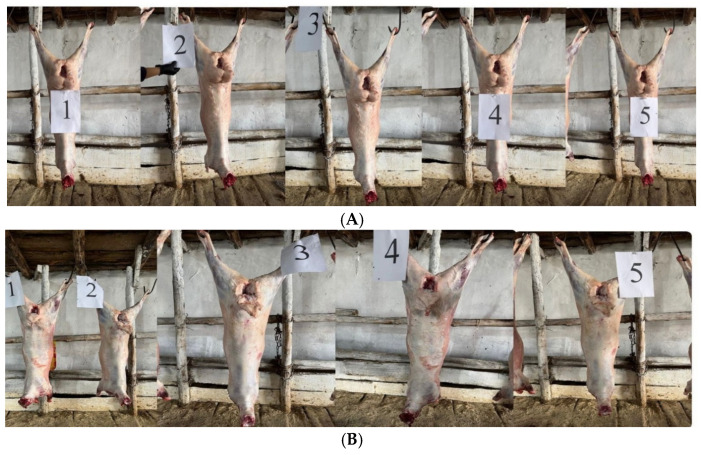
Meat-type carcass conformation of ram lambs at 4–4.5 months of age: (**A**) control group (SCW × SCW); (**B**) experimental group (MW × SCW).

Tail fat weight showed the greatest breed-related variation (one-way ANOVA: F (3,16) = 561.00; *p* < 0.001). Tail fat was present only in the fat-tailed genotypes, namely CW (2.1 ± 0.16 kg) and SCW (2.0 ± 0.16 kg), whereas it was absent in the MM × CW and MW × SCW lambs. Consequently, the crossbred animals were characterized by a more pronounced meat-type carcass conformation and reduced fat deposition in the tail region (Figure 4).

Overall, the MW × SCW genotype exhibited the highest pre-slaughter live weight, carcass weight, slaughter weight, and internal fat weight among the evaluated genotypes, whereas the crossbred groups were characterized by the absence of tail fat.

### 3.3. Morphological Composition of Carcasses

Significant differences were observed in all components of carcass morphological composition (muscle, fat, and bone tissues) (*p* < 0.001). The morphological compositions of the carcasses are presented in Table 3.

Carcass weight was significantly affected by genotype (ANOVA: F (3,16) = 11.49; *p* < 0.001). The highest carcass weight was recorded in rams of the MW × SCW genotype (24.0 ± 0.72 kg), which exceeded the purebred CW group (20.0 ± 1.30 kg) by 20.0%. According to Tukey’s post hoc test, the MW × SCW group had significantly higher carcass weight than the CW and SCW groups (*p* < 0.05), whereas the MM × CW group also exceeded the CW group (*p* < 0.05). No significant differences were detected between the two crossbred groups or between the two purebred groups (*p* > 0.05).

Lean meat weight also differed significantly among genotypes (ANOVA: F (3,16) = 21.78; *p* < 0.001). Crossbred lambs (MM × CW and MW × SCW) produced significantly greater lean meat weights than the corresponding purebred CW and SCW groups (*p* < 0.05). No significant differences were detected between the two crossbred genotypes or between the two purebred genotypes (*p* > 0.05).

Bone weight was significantly influenced by genotype (ANOVA: F (3,16) = 4.88; *p* = 0.013). The CW group had significantly higher bone weight than the MM × CW and MW × SCW groups (*p* < 0.05), whereas the SCW group showed intermediate values and did not differ significantly from either the CW or the crossbred groups (*p* > 0.05).

### 3.4. Meatiness Coefficient

**Figure 5:**
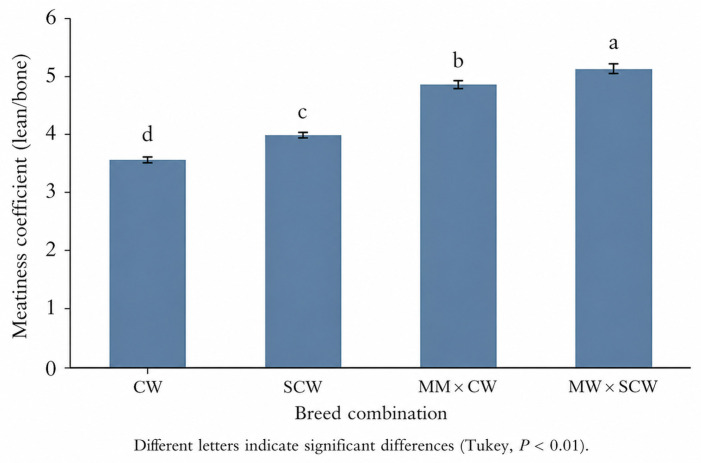
Meatiness coefficient of ram lambs aged 4–4.5 months. Note: Different superscript letters indicate significant differences between means according to Tukey’s post hoc test (*p* < 0.01).

The meatiness coefficient differed significantly among the experimental groups (one-way ANOVA: F = 2506.60; df = 3,16; *p* < 0.001), as shown in Figure 5. The recorded values were as follows:

- SCW × MW: 5.15 ± 0.04 ^a^;
- CW × MM: 4.89 ± 0.05 ^b^;
- SCW: 4.05 ± 0.02 ^c^;
- CW: 3.57 ± 0.01 ^d^.

The Tukey post hoc test confirmed significant differences between all pairwise comparisons of the four genotypes (*p* < 0.01). The MW × SCW group exhibited the highest meatiness coefficient and significantly outperformed the MM × CW, SCW, and CW groups. Likewise, the MM × CW group showed significantly higher values than both purebred groups (SCW and CW), while the SCW group also had a significantly higher meatiness coefficient than the CW group.

Crossbred rams exhibited meatiness coefficients that were 21–44% higher than those of the purebred groups, reflecting a greater muscle-to-bone ratio. The highest meatiness coefficient was recorded in the MW × SCW genotype.

## 4. Discussion

The results of this study demonstrate that crossbreeding significantly enhances the meat productivity of Kazakh sheep under natural pasture conditions without supplementary feeding. Crossbred rams (MM × CW and MW × SCW) outperformed purebred counterparts in live weight, average daily gains, carcass weight, slaughter yield, and meatiness coefficient.

### 4.1. Growth Performance Under Pasture Conditions

Significant differences in birth live weight were observed among the experimental groups (one-way ANOVA: F = 34.95; df = 3116; *p* < 0.001), with the MW × SCW genotype showing the highest value (5.03 kg). According to Tukey’s post hoc test, the MW × SCW lambs had significantly higher birth live weight than all other genotypes (*p* < 0.05), whereas the SCW × SCW and CW groups did not differ significantly from each other.

The higher birth weight of crossbred lambs most likely reflects favorable additive and non-additive genetic effects inherited from the terminal sire breeds. This early developmental advantage is important because birth weight is positively associated with postnatal growth, survival, and subsequent slaughter performance under extensive production systems.

This finding is particularly noteworthy because all lambs were raised exclusively on natural pastures with varying degrees of degradation (forage yield 0.3–5.0 c/ha) and without supplementary feeding. The higher birth live weight of the MW × SCW lambs, combined with their low variability (CV = 4.57%), suggests favorable maternal and direct genetic effects [24,25,26].

Although live weight at 4–4.5 months tended to be higher in the crossbred groups (38.20–38.40 kg) than in the purebred CW animals (36.70 kg), the differences were not statistically significant (one-way ANOVA: F = 2.39; df = 3116; *p* = 0.072). Nevertheless, significant differences were detected for absolute weight gain (F = 2.98; df = 3116; *p* = 0.034) and average daily gain (F = 2.98; df = 3116; *p* = 0.034). According to Tukey’s post hoc test, the CW × CW group showed significantly lower absolute and average daily weight gains than the MM × CW and SCW × SCW groups (*p* < 0.05), whereas the MW × SCW group occupied an intermediate position and did not differ significantly from either the crossbred or purebred groups.

The average daily gains obtained in this study (267–283 g/day) are particularly noteworthy considering that all lambs were raised exclusively on natural pastures, which in the study area exhibited signs of degradation, including reduced vegetation cover and the replacement of valuable feather-grass communities by less productive wormwood–fescue associations. Despite these challenging feeding conditions, the crossbred lambs maintained growth rates comparable to or higher than those of the purebred groups.

These findings indicate that genetic improvement through crossbreeding can partially compensate for limitations in pasture quality by improving the biological efficiency of growth rather than by increasing feed inputs. This observation is particularly relevant for low-input sheep production systems, where opportunities to improve nutrition are often limited by environmental and economic constraints.

### 4.2. Slaughter Traits and Meat Quality

The sample slaughter of five rams from each group revealed highly significant differences in all carcass traits (*p* < 0.001). A slaughter yield of 54.0% achieved in both crossbred groups is competitive with international standards reported for specialized meat sheep raised under semi-intensive production systems [27]. Considering that the lambs in the present study were raised exclusively on natural pastures without supplementary feeding, these values indicate efficient conversion of pasture resources into saleable carcass weight.

The absence of tail fat in crossbred rams, combined with increased internal fat deposition, represents a desirable shift in fat distribution toward a meat-type sheep conformation. Tail fat is characteristic of fat-tailed breeds adapted to arid conditions but is considered less desirable in modern meat production due to reduced dressing percentage and consumer preferences [28].

From a biological perspective, the reduction in tail fat suggests that a greater proportion of available nutrients was directed toward muscle development rather than the accumulation of fat reserves in the tail. This redistribution contributes to improved carcass composition without compromising the adaptive capacity inherited from the local maternal breeds.

The crossbreeding strategy effectively reduced tail fat while maintaining adequate fat reserves through internal fat deposition, improving both carcass conformation and commercial value.

Internal fat remains an important physiological energy reserve and contributes to animal adaptation under extensive grazing conditions. Therefore, the observed increase in internal fat, accompanied by the elimination of tail fat, may be regarded as a more economically favorable pattern of fat deposition for modern meat production.

This is particularly relevant for the global market, where consumer preferences in both developing and developed countries are shifting toward leaner meat with moderate marbling [29].

Overall, the slaughter characteristics observed in the MW × SCW and MM × CW genotypes demonstrate that terminal crossbreeding can simultaneously improve carcass yield and optimize carcass composition without increasing feeding intensity. These findings support the application of specialized meat sires as an effective breeding strategy for extensive sheep production systems in arid and semi-arid environments.

### 4.3. Carcass Composition and Meatiness Coefficient

The meatiness coefficient values obtained in this study (3.57–5.15) demonstrated significant genetic differences (one-way ANOVA: F = 2506.60; df = 3,16; *p* < 0.001). The highest value (5.15) was recorded in the MW × SCW crossbred lambs, indicating superior muscularity and carcass composition comparable to those reported for specialized meat breeds such as Suffolk and Texel [30].

The meatiness coefficient is considered one of the most informative indicators of carcass quality because it reflects the relationship between edible muscle tissue and the non-edible skeletal component. Therefore, an increase in this coefficient directly indicates improved biological and commercial efficiency of meat production.

Similar meatiness coefficient values (4.8–5.2) have been reported for Awassi × Dorper crosses in Jordan and Merino × Dorper crosses in South Africa [12,13], confirming the effectiveness of terminal crossbreeding for improving carcass composition under arid and semi-arid conditions.

The superiority of the MW × SCW genotype is most likely associated with the successful combination of two complementary genetic characteristics: the excellent adaptation of Kazakh Semi-coarse-wooled ewes to local environmental conditions and the high genetic potential for muscle development inherited from Kazakh Meat-Wool rams. Such complementarity allows crossbred animals to express heterosis while maintaining adaptation to harsh grazing environments.

The significant reduction in bone percentage in crossbred lambs (16.2–17.0%) compared with purebred animals (19.9–22.0%) directly increases carcass value because bone tissue represents a non-marketable component of the carcass.

At the same time, the higher proportion of lean meat (up to 83.8%) demonstrates that a larger fraction of carcass weight consisted of commercially valuable muscle tissue. This improvement is particularly important for processors and consumers, since lean meat determines both carcass value and processing efficiency.

The fact that these improvements were achieved under natural grazing conditions without concentrate supplementation indicates that genetic improvement can substantially increase carcass quality even where nutritional resources are limited.

From a practical breeding perspective, these findings demonstrate that selection based on crossbreeding with specialized meat sires can improve carcass composition more efficiently than environmental improvements alone, making crossbreeding one of the most practical genetic tools for extensive sheep production. Consequently, this breeding strategy represents a practical and economically sustainable approach for increasing meat production in extensive sheep farming systems.

### 4.4. Comparative Context: Global Significance of the Results

The magnitude of heterosis observed in this study, reflected by the 10–25% increase in the meatiness coefficient, lean meat yield of up to 83.8%, and slaughter yield of 54.0%, is consistent with results reported for terminal crossbreeding programs involving fat-tailed sheep populations in other arid and semi-arid regions of the world.

For example, Awassi × Dorper crosses in Jordan achieved slaughter yields ranging from 52.5% to 55.3% and muscle tissue yields of approximately 82.4% [12]. Likewise, Merino × Dorper crosses in South Africa reached a slaughter yield of 53.2% and a meatiness coefficient of 4.95 [13]. In Tunisia, crossing Barbarine sheep with terminal meat breeds increased slaughter yield from 47.8% to 53.6% [14].

The close agreement between the present findings and those reported from geographically distant production systems suggests that the beneficial effects of terminal crossbreeding are largely independent of regional differences and can be consistently expressed under diverse pasture-based production environments. This consistency strengthens the evidence that crossbreeding represents a reliable genetic strategy for improving meat productivity under extensive management conditions.

An important feature of the present study is that comparable productivity was achieved under natural pasture conditions without concentrate supplementation. This demonstrates that substantial improvements in carcass characteristics can be obtained through genetic improvement alone, even where opportunities for nutritional intensification are limited.

The similarity of results from production systems which are geographically distant but ecologically comparable highlights the universal potential of crossbreeding with terminal sires under low-input conditions. This has direct relevance for the following regions:

- Central Asian countries (Kazakhstan, Kyrgyzstan, Uzbekistan, Turkmenistan), where sheep production remains a cornerstone of rural livelihoods [31,32];
- Mongolia and Inner Mongolia (China), where pastoral systems face similar challenges of pasture degradation and climatic variability [33];
- Sub-Saharan Africa, where fat-tailed and fat-rumped sheep breeds dominate and the use of terminal sires is limited [34,35];
- The Middle East and North Africa (MENA) region, where breeds such as Awassi could benefit from similar crossbreeding schemes [12,13,36].

Overall, the consistency of these results across different countries supports the broader applicability of terminal crossbreeding as a practical approach for improving meat production in arid and semi-arid regions worldwide.

### 4.5. Significance for Pasture-Based Systems

Pasture monitoring conducted during the present study demonstrated progressive degradation of natural grazing lands caused by long-term uncontrolled grazing. The replacement of productive feather-grass–fescue communities by less productive wormwood–fescue associations, together with the decline in pasture productivity to as little as 0.3 c/ha in severely degraded areas, created nutritionally challenging conditions for lamb growth.

Despite these limitations, crossbred lambs consistently demonstrated superior growth performance, carcass weight, slaughter yield, and meatiness coefficient compared with the corresponding purebred groups. These findings indicate that crossbreeding can partially compensate for nutritional constraints by improving the biological efficiency of feed utilization under extensive grazing conditions.

The results also suggest that genetic improvement represents one of the most economically feasible approaches for increasing meat production in low-input sheep farming systems, where the use of concentrate feeds and intensive management is often limited by financial constraints.

However, genetic improvement should not be considered a substitute for sustainable pasture management. Maximum expression of the genetic potential of crossbred animals requires appropriate grazing management practices, including controlled stocking rates, rotational grazing, and restoration of degraded pasture areas. The integration of genetic improvement with sustainable pasture utilization is consistent with current international recommendations for livestock production in arid and semi-arid regions [37].

Our results suggest that genetic improvement through crossbreeding can substantially improve meat productivity under pasture-based conditions without relying on capital-intensive feed supplementation or imported concentrates, making this approach particularly relevant for small-scale sheep farming in arid and semi-arid regions.

### 4.6. Limitations and Future Research Directions

Although the present study demonstrated clear advantages of crossbreeding under practical pasture conditions, several limitations should be acknowledged.

The experiment was conducted under natural grazing conditions without supplementary feeding in order to reflect commercial sheep production systems in Kazakhstan. Therefore, the obtained results characterize the productive performance of different genotypes under extensive management conditions.

Although pasture vegetation and botanical composition were evaluated, the chemical composition of the forage was not determined. Future studies should combine nutritional characterization of pasture resources with productivity assessment to better understand genotype × environment interactions under extensive grazing systems.

In addition, the present study evaluated growth performance and carcass traits of ram lambs only up to 4–4.5 months of age. Further research should assess the long-term productive performance, reproductive traits, and economic efficiency of crossbreeding under different environmental conditions.

Validation of these breeding strategies across different ecological zones of Central Asia would further strengthen the applicability of the proposed breeding approach.

## 5. Conclusions

The present study demonstrated that crossbreeding is an effective genetic strategy for improving meat productivity of sheep under extensive pasture-based production systems in northeastern Kazakhstan. Significant differences were observed among the four genotype groups in growth performance, slaughter characteristics, carcass composition, and meatiness coefficient.

The crossbred genotypes (MM × CW and MW × SCW) showed superior slaughter performance and carcass quality compared with the corresponding purebred groups. They produced heavier carcasses, higher slaughter yields (54.0%), greater proportions of lean meat (83.0–83.8%), and significantly higher meatiness coefficients (4.90–5.15), while reducing tail fat deposition and improving carcass conformation.

These advantages were achieved under practical commercial grazing conditions without supplementary feeding, indicating that crossbreeding can substantially improve meat production efficiency even under the nutritional limitations typical of extensive sheep farming.

The obtained results support the use of terminal sire crossbreeding programs for improving lamb meat production in low-input grazing systems of Kazakhstan and other arid and semi-arid regions with similar environmental conditions.

Further studies should evaluate the long-term productive performance, reproductive traits, and economic efficiency of these crossbreeding systems under different production environments.

## Figures and Tables

**Table 1:** Live weights and live weight gains of ram lambs from birth to 4–4.5 months (*n* = 30 per group).

Farm	Breed/Genotype	Birth Live Weight (kg)	Live Weight at 4–4.5 Months (kg)	Absolute Gain (kg)	Average Daily Gain (g)
LLP “Ak Bas-Pavlodar”	CW × CW	4.70 ± 0.25 ^b^	36.70 ± 1.79 ^a^	32.00 ± 1.86 ^b^	267 ± 15 ^b^
	MM × CW	4.30 ± 0.41 ^c^	38.20 ± 3.05 ^a^	33.90 ± 3.02 ^a^	282 ± 32 ^a^
Peasant Farm “Bepen”	SCW× SCW	4.41 ± 0.30 ^bc^	38.33 ± 2.23 ^a^	33.92 ± 2.25 ^a^	283 ± 19 ^a^
	MW × SCW	5.03 ± 0.23 ^a^	38.40 ± 3.92 ^a^	33.37 ± 3.88 ^ab^	278 ± 25 ^ab^

Note: Different superscript letters within the same column indicate significant differences between means according to Tukey’s post hoc test (*p* < 0.05). One-way ANOVA showed significant effects of genotype on birth live weight (F = 34.95; df = 3116; *p* < 0.001), absolute weight gain (F = 2.98; df = 3116; *p* = 0.034), and average daily gain (F = 2.98; df = 3116; *p* = 0.034), while no significant differences were observed for live weight at 4–4.5 months (F = 2.39; df = 3116; *p* = 0.072).

**Table 2:** Slaughter characteristics of rams from sample slaughter at 4–4.5 months of age (*n* = 5 per group).

Breed	Farm	Pre-Slaughter Live Weight, kg	Carcass Weight		Tail Fat Weight		Internal Fat Weight		Slaughter Weight	
			kg	%	kg	%	kg	%	kg	%
CW	LLP “Ak Bas-Pavlodar”	39.4 ± 1.10 ^c^	20.0 ± 1.30 ^b^	50.8	2.1 ± 0.16 ^a^	5.3	0.30 ± 0.07 ^c^	0.7	20.3 ± 1.25 ^b^	51.5
MM × CW		43.5 ± 1.61 ^ab^	23.0 ± 1.22 ^a^	52.8	–	–	0.50 ± 0.07 ^b^	1.1	23.5 ± 1.20 ^a^	54.0
SCW	Peasant Farm “Bepen”	42.5 ± 1.04 ^b^	21.0 ± 1.44 ^b^	49.4	2.0 ± 0.16 ^a^	4.7	0.25 ± 0.05 ^c^	0.5	21.2 ± 1.45 ^b^	49.9
MW × SCW		46.0 ± 2.17 ^a^	24.0 ± 0.72 ^a^	52.1	–	–	0.80 ± 0.07 ^a^	1.7	24.8 ± 0.70 ^a^	54.0

Note: Different superscript letters within the same column indicate significant differences between means according to Tukey’s post hoc test (*p* < 0.05). One-way ANOVA revealed significant effects of genotype on pre-slaughter live weight (F = 15.52; df = 3,16; *p* < 0.001), carcass weight (F = 11.49; df = 3,16; *p* < 0.001), tail fat weight (F = 561.00; df = 3,16; *p* < 0.001), internal fat weight (F = 72.43; df = 3,16; *p* < 0.001), and slaughter weight (F = 15.14; df = 3,16; *p* < 0.001).

**Table 3:** Morphological composition of carcasses of rams from the sample slaughter at 4–4.5 months of age (*n* = 5 per group).

Breed	Carcass Weight, kg	Lean Meat		Bones	
		kg	%	kg	%
CW	20.0 ± 1.30 ^b^	15.6 ± 1.02 ^b^	78	4.4 ± 0.29 ^a^	22
MM × CW	23.0 ± 1.22 ^a^	19.1 ± 1.03 ^a^	83	3.9 ± 0.22 ^b^	17
SCW	21.0 ± 1.44 ^b^	16.8 ± 1.17 ^b^	80.1	4.2 ± 0.27 ^ab^	19.9
MW × SCW	24.0 ± 0.72 ^a^	20.1 ± 0.60 ^a^	83.8	3.9 ± 0.12 ^b^	16.2

Note: Different superscript letters within the same column indicate significant differences between means according to Tukey’s post hoc test (*p* < 0.05). One-way ANOVA revealed significant effects of genotype on carcass weight (F = 11.49; df = 3,16; *p* < 0.001), boneless meat weight (F = 21.78; df = 3,16; *p* < 0.001), and bone weight (F = 4.88; df = 3,16; *p* = 0.013).

## Data Availability

The data presented in this study are available from the corresponding author upon reasonable request.
